# Artificial intelligence-assisted reduction in patients’ waiting time for outpatient process: a retrospective cohort study

**DOI:** 10.1186/s12913-021-06248-z

**Published:** 2021-03-17

**Authors:** Xiaoqing Li, Dan Tian, Weihua Li, Bin Dong, Hansong Wang, Jiajun Yuan, Biru Li, Lei Shi, Xulin Lin, Liebin Zhao, Shijian Liu

**Affiliations:** 1 School of Public Health, Shanghai Jiao Tong University School of Medicine, Shanghai, China; 2 Child Health Advocacy Institute, Shanghai Children’s Medical Center, Shanghai Jiao Tong University School of Medicine, 1678 Dongfang Road, Shanghai, 200127 China; 3 Division of Hospital Management, Shanghai Children’s Medical Center, Shanghai Jiao Tong University School of Medicine, 1678 Dongfang Road, Shanghai, 200127 China; 4 Pediatric AI clinical Application and Research Center, Shanghai Children’s Medical Center, Shanghai, China; 5 Shanghai Engineering Research Center of Intelligence Pediatrics (SERCIP), Shanghai, China; 6 Child Health Advocacy Institute, China Hospital Development Institute of Shanghai Jiao Tong University, Shanghai, China; 7 Department of Pediatric Internal Medicine, Shanghai Children’s Medical Center, Shanghai Jiao Tong University School of Medicine, Shanghai, China; 8 Hangzhou YI TU Healthcare Technology CO. Ltd, Hangzhou, China

**Keywords:** Artificial intelligence, Outpatient, Waiting time, Medical system

## Abstract

**Background:**

Many studies suggest that patient satisfaction is significantly negatively correlated with the waiting time. A well-designed healthcare system should not keep patients waiting too long for an appointment and consultation. However, in China, patients spend notable time waiting, and the actual time spent on diagnosis and treatment in the consulting room is comparatively less.

**Methods:**

We developed an artificial intelligence (AI)-assisted module and name it XIAO YI. It could help outpatients automatically order imaging examinations or laboratory tests based on their chief complaints. Thus, outpatients could get examined or tested before they went to see the doctor. People who saw the doctor in the traditional way were allocated to the conventional group, and those who used XIAO YI were assigned to the AI-assisted group. We conducted a retrospective cohort study from August 1, 2019 to January 31, 2020. Propensity score matching was used to balance the confounding factor between the two groups. And waiting time was defined as the time from registration to preparation for laboratory tests or imaging examinations. The total cost included the registration fee, test fee, examination fee, and drug fee. We used Wilcoxon rank-sum test to compare the differences in time and cost. The statistical significance level was set at 0.05 for two sides.

**Results:**

Twelve thousand and three hundred forty-two visits were recruited, consisting of 6171 visits in the conventional group and 6171 visits in the AI-assisted group. The median waiting time was 0.38 (interquartile range: 0.20, 1.33) hours for the AI-assisted group compared with 1.97 (0.76, 3.48) hours for the conventional group (*p* < 0.05). The total cost was 335.97 (interquartile range: 244.80, 437.60) CNY (Chinese Yuan) for the AI-assisted group and 364.58 (249.70, 497.76) CNY for the conventional group (*p* < 0.05).

**Conclusions:**

Using XIAO YI can significantly reduce the waiting time of patients, and thus, improve the outpatient service process of hospitals.

## Background

Global population explosion and increasing life expectancy have led to a surge in patients seeking medical services. When the medical demand exceeds a hospital’s capacity, the patients’ waiting time is prolonged [1]. Waiting time in outpatient clinics is recognized as one of the main issues in outpatient healthcare worldwide [2]. It has two dimensions: actual waiting time and perceived waiting time [3]. Some studies indicate that patient satisfaction is significantly negatively correlated with actual waiting time [2, 4–7]. While some studies believe the perception towards waiting time will affect overall satisfaction, but actual waiting time will not [3, 8]. Table 1 introduces research on waiting times. But all in all, a well-functioning hospital ideally should not keep patients waiting too long for appointment and consultation [2, 9].

**Table 1:** Literature review and summary on perception waiting time and actual waiting time

Study	Year	Outcome	Conclusions
Thompson DA, et al. [3]	1996	PWT	Satisfaction depended more on PWT than AWT.
Gartner D, et al. [8]	2020	PWT	Reducing PWT could potentially improve patient satisfaction.
Sun J, et al. [2]	2017	AWT	Reducing AWT could improve patient satisfaction.
Michael M, et al. [4]	2013	AWT	Significant reductions in AWT was observed along with an increase in patient satisfaction.
Xie Z, et al. [5]	2017	AWT	AWT was negatively associated with patient satisfaction.
Xie W, et al. [6]	2019	AWT	The reservation service shortened patient’s AWT and improved patient satisfaction.
Liu J, et al. [7]	2019	AWT	Outpatients’ overall satisfaction was associated with AWT.

*PWT* Perception Waiting Time, *AWT* Actual Waiting Time

In China, outpatients need to wait for a considerable amount of time, whereas the actual time spent on diagnosis and treatment in the consulting room is comparatively very short. There are two main reasons for this. First, most Chinese hospitals do not require patients to have a prescheduled appointment [6]. Most patients wait to see a doctor on the day of their registration. Considering China has more than 1.4 billion people but fewer than 5 million doctors, it is conceivable that every doctor’s availability is fully booked, especially in the tertiary hospitals. The second reason for hospital overcrowding is the imperfect family doctor appointment system. In Europe and North America, family doctors resolve residents’ common illnesses, and they establish long-term service relationships with patients and their families [10]. The Chinese government has implemented a three-tier system. The primary hospital is responsible for basic needs and common diseases. For issues beyond the primary hospital’s capabilities, the patient is referred to a secondary hospital and then to a tertiary hospital as necessary. However, the system is not mandatory, and the patients’ choices are respected. Even if their conditions are likely to be resolved by primary or secondary hospitals, patients prefer tertiary general hospitals because of their better medical equipment and specialists [4].

Because of the large number of pediatric outpatient clinics and a brain drain of pediatricians in recent years, these problems are particularly prominent in pediatric hospitals. Therefore, it is of great practical significance to analyze the queuing process and simplify the outpatient procedure in order to reduce the waiting time. For this purpose, the use of artificial intelligence (AI) is worth exploring. AI-based methods have emerged as powerful tools to transform medical care. In a retrospective study conducted by the team of Guangzhou Women and Children’s Medical Center and Hangzhou YI TU Healthcare Technology Co. Ltd., AI enabled high diagnostic accuracy for common diseases comparable with pediatricians [11]. They developed a natural language processing (NLP) model based on deep learning to extract clinical information from electronic medical record, and then built a diagnostic system based on the extracted features. So, AI could generate its own diagnosis, much like a human doctor’s clinical reasoning process. And in our collaboration with YI TU, we used a similar modeling approach, drawing on their previous success. In addition, AI has also been applied for emergency room and laboratory (lab) procedures; it showed strong performance in predicting waiting times and optimizing processes [12–16]. The emergency appointment systems in Europe and the United States are highly similar to the Chinese outpatient system, as they do not require advance appointments [17].

With this background, we propose an AI-assisted approach for improving the efficiency of the outpatient service. In this study, we applied AI to the existing system of the Shanghai Children’s Medical Center (SCMC) to diagnose patients in advance and recommend examinations or tests for the patients. Patients took the examinations or tests before seeing a doctor, which reduced their waiting time. And we studied the impacts of this AI-assisted approach on patients’ waiting time and expenses.

## Methods

### Establishment of the AI model

Based on deep learning, the SCMC and YI TU Technology Company jointly developed a personalized inquisition and automatic diagnosis algorithm that could mimic the consultation with a doctor. In the first place, the Electronic Medical Records (EMRs) were structured through Natural language processing (NLP). We selected 59,041 high-quality EMRs which were manually noted by professional doctors and informatics experts. This NLP model utilized deep learning to automate free texts from EMRs into standardized clinical features, allowing further processing of clinical information for diagnostic classification. Logistic regression classifiers were used to establish a hierarchical diagnostic system, and the system was primarily based on anatomic divisions. Following by automatic diagnosis based on medical records, the corresponding examinations or tests items were generated. This approach, which integrated the functions of inquiry, medical history collection, diagnosis and ordering tests or examinations, had been put into use, and we named it XIAO YI. The algorithm was similar to that of Liang’s [11], except that our model had been updated and iterated base on the data from our hospital information system. Besides, in Liang’s study, they focused on using AI to diagnose pediatric diseases, but our study used AI to prescribe examinations and tests before seeing a doctor to reduce the waiting time of patients in hospital lines.

Considering guardian’s acceptance, though AI algorithm could theoretically create most of the tests/examinations, our final client-side only considered certain kinds of the tests/examinations, which were noninvasive (or less invasive) and low-cost. This was currently set in the backstage, and no additional manual operation was needed. Thus, XIAO YI just recommended common items to patients. If a 12-year-old child urinated blood with lumbago for 1 day, the first diagnosis might be kidney stones. According to the inquisition, XIAO YI analyzed the child needed blood routine, urine routine and urinary B-ultrasound. But in some cases, doctors might also ask the patient to have a CT scan. The price of CT was higher, but B-ultrasound was sufficient for a preliminary diagnosis of kidney stones. In performance test, most errors were items missing (85%). This was the result of our deliberate choice, as we did not require XIAO YI to order all tests/examinations for patients. On the contrary, we only needed it to issue the simplest and most common parts. The rest of the complex, invasive ones would be left to professional doctors.

At the same time, in each department, we also had special backstage doctors responsible for reviewing every item ordered by XIAO YI. The doctors would adjust the tests/examinations manually according to the actual condition. For example, some parents wanted to add other tests/examinations that were not related to the disease. That didn’t happen often, though. Only after the doctors’ approval, can the patients pay and complete the tests/examinations.

### Procedure of the AI-assisted outpatient service

We explain the standard outpatient service process and the AI-based modifications to it. In the traditional way, patients need to register first, and after registration they will wait in the waiting area. When it is their turn, they go to the consulting room to see a doctor. Mostly, a lab test or an imaging examination is needed to confirm the diagnosis. And then patients have to pay for these, and go to the correct places to get examined or tested. After receiving the reports, patients will wait again to see the doctor and may be recommended another examination/test or some medicines. In this study, we focus on the steps from registration to the examination or test.

**Fig. 1:**
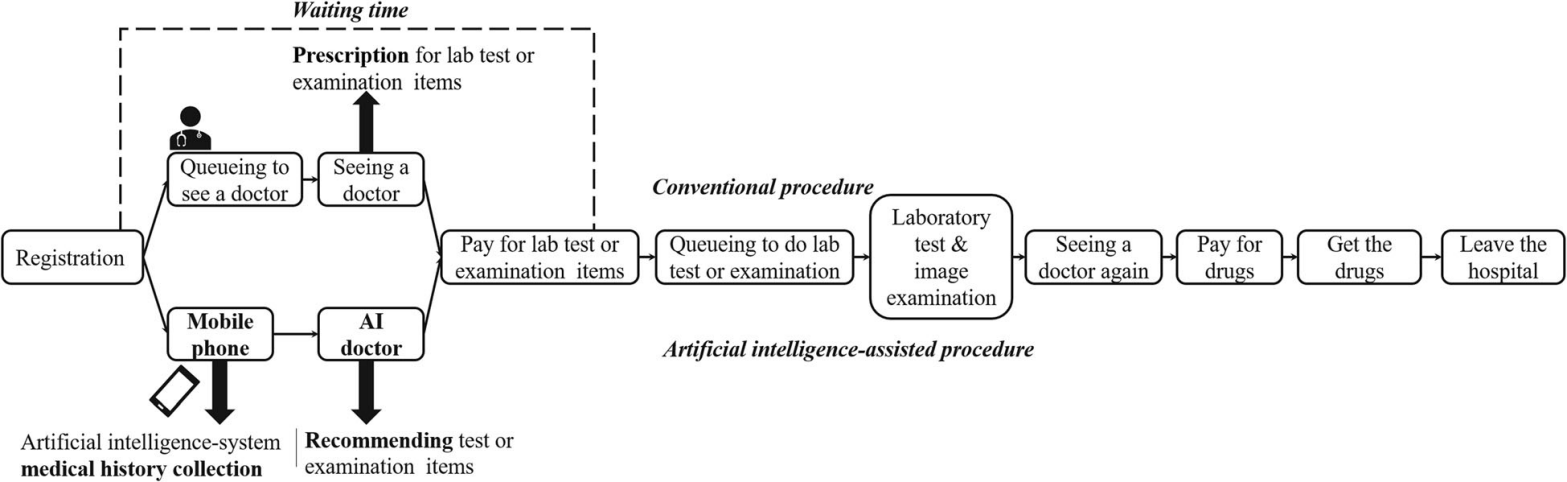
The process of outpatient in Shanghai Children’s Medical Center

The first step in the AI-assisted outpatient service is registration, too. In the next step, patients click the WeChat application (a WhatsApp-like social application widely used in China) on their mobile phone. Patients’ unique outpatient numbers are linked to a small smart program based on WeChat, that is XIAO YI client-side. XIAO YI client-side is the materialization of the above-discussed algorithms, which has clients on both mobile phones and doctors’ working computers. It automatically reads the registration information of patients. Depending on the chief complaint, XIAO YI asks the patients a series of questions, like a real doctor would do. The next question is decided intelligently based on the answer to the previous question. When XIAO YI believes it has gathered enough information, the inquisition ends. XIAO YI orders tests or examinations that must be done to help the doctors make the clinical diagnosis. The tests and examinations “prescribed” by XIAO YI are basic, minor trauma, and relatively inexpensive (e.g., blood routine). Patient then make the payment for these tests and head to the testing rooms. If patients disagree, they would go through the traditional process of waiting in line to see the human doctor. When the test or examination is completed and the report is obtained, patients wait to be called to the doctor’s office for consultation. The traditional and AI-assisted workflows are shown in Fig. 1.

### Selection of subjects

SCMC is one of the biggest pediatric specialized hospitals in Shanghai. It affiliates to Shanghai Jiao Tong University School of Medicine. We collected information of patient’s registrations from August 1, 2019 to January 31, 2020. The dataset included patients from the internal department, gastroenterology department, and respiratory department who visited SCMC during that period. It included their sex, age (on the day of registration), registration code, registration time, time of meeting the doctor, time of examination/testing, time of prescription by the doctor, and time of receiving the medicines, among others. We ensured patients’ privacy. In the dataset that we extracted and used for analysis, researchers could not see the patient’s name or their outpatient number. The patient’s outpatient number was recoded into a registration code, mainly because sometimes a patient would register multiple times in one day and therefore the outpatient number needed to be recoded to make it unique. In addition, in this way, the information security of patients was also guaranteed.

During this period, uniformly trained volunteers and nurses would publicize XIAO YI to the guardians of children in the internal department, gastroenterology department, and respiratory department, and directed them how to use it. With the help of volunteers, some guardians used XIAO YI to order and complete tests/examinations before they went to see a doctor, while some guardians sticked to the traditional way of seeing a doctor. Thus, patients were classified into two groups, namely, the conventional outpatient group and the AI-assisted group (AI group), depending on their own choices. Because the outpatient service process selected by patients was equivalent to exposure, and the length of the waiting time was equivalent to outcome, so we conducted a retrospective cohort study. The two groups of patients were matched first according to the registration time mainly because the time of registration might be the most influential factor affecting the waiting time of an outpatient except the grouping. Generally, there are more patients on holidays than on weekdays, and there are more patients in the morning than in the afternoon. Moreover, weather, traffic jam, and other external factors (e.g., COVID-19 outbreak) could influence the time spent by outpatients in the hospital. We needed to reduce the interference of other factors with the results, thus, we paired the patients who visited the hospital at almost the same time. And propensity score matching (PSM) was employed to balance this covariate [18].

We found that using only the paired dataset was insufficient. This was because in our conceptual scenario, patients were first registered, signed in and then queued up in the waiting area to see the doctors. However, the actual situation was that after registration, they did not sign in at once if they perceived there was a long waiting time due to too many patients. They (i.e., children accompanied by their guardians) might wait until there were fewer patients before signing in and waiting to see a doctor. As a result, this kind of patients spent much more waiting time than others. In addition, there were some patients who took advantage of the features of the system to make an appointment, especially in the AI group, as it was more convenient to make an appointment through the AI system. For example, if a patient came to register at 8 a.m. but the patient was not available until 2 p.m., the patient would request the nurse to schedule the appointment for 2 p.m. This would greatly overestimate the time spent in the hospital.

To avoid these issues, we cleaned the data according to some criteria. We excluded patients who did not have a lab test because the main function of the AI was to order a lab test before the consultation with doctor. Patients who spent more than 5 h from registration to consultation were also excluded, as were those who spent more than 8 h from registration to obtain their medicines. According to the experience of many doctors in the hospital, such long waiting times usually happened because the patients either had appointment or were late for their appointment. The patients who spent less than 5 min waiting were also excluded, as these were likely errors.

### Outcomes

The primary outcome was the time spent by the patient from registration to take the laboratory test or examination, defined as the waiting time. The secondary outcome was the expenses in the hospital. Thus, we evaluated the performance of the AI-system from two dimensions. In addition, patients in the AI-assisted group and the conventional group were subdivided into six subgroups according to three clinic departments, including internal department, gastroenterology department and respiratory department to further analyze the waiting times. Besides, patients were also subdivided depending on the types of tests (blood routine test, routine urine test and detection of influenza A and B virus test) or examinations (abdomen ultrasound and chest radiograph).

### Statistical analysis

Stata 15 was used for statistical analysis and PSM. Continuous variables were expressed as means ± standard deviation (SD) or medians and inter-quartile range (IQR). Categorical variables were summarized as counts and percentages. Missing data were not imputed and deleted. All of the analyses were two-sided, and *P* values of < 0.05 were considered to be significant. The skewness/kurtosis test for normality was used to test the assumption of normal distribution. When normally distributed, continuous variables were expressed as mean ± SD and calculated using a paired Student’s *t*-test. If not, as was the case with almost all continuous variables, we used the nonparametric Wilcoxon signed-rank test.

Propensity scores were estimated using logistic regression. The covariate was time of registration. This covariate was selected because it might affect the time that the patient spent in the hospital. The time from registration to take the test or examination was entered into the regression model as a dependent variable. The group was defined as an independent variable. A 1:1 nearest neighbor, case-control match without replacement was used [19]. Stata was used to test the equilibrium between the two groups after PSM, and *p* > 0.05 suggested that the difference in registration time was not statistically significant. The chi-square test was used to compare the sex ratio in the two groups and the ratio of visits in each department.

## Results

### Data preparation and model validation

**Fig. 2:**
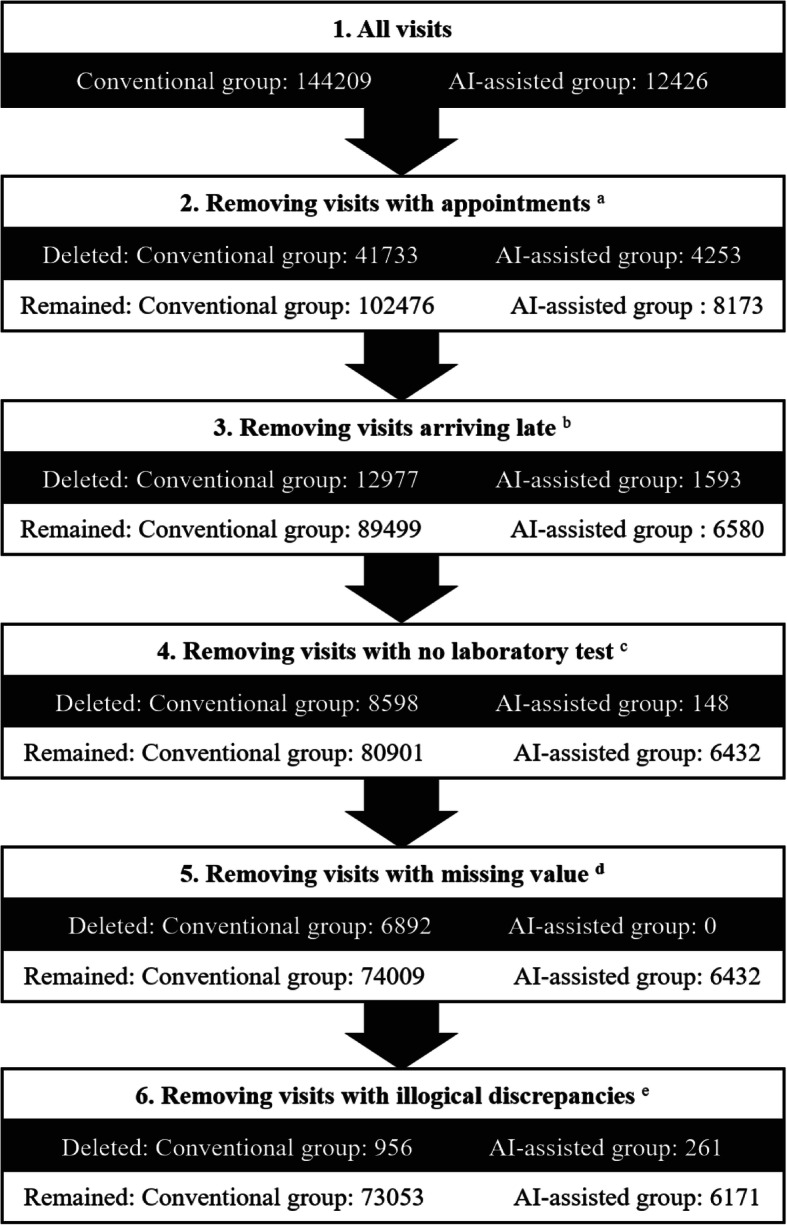
Procedures of data cleaning. **a** A few observations had appointments, who might come very late after registration, were excluded from our analysis. **b** We also discarded patients arriving late to prevent them from interfering with the result. **c** Since this study mainly shortened the time of the patients undergoing the laboratory test, the patients who did not have the test should be deleted. **d** The data of some patients recorded the time they got their medicine but the corresponding drug costs were missing. This part of data was deleted as missing value.**e** patients with illogical discrepancies was deleted. For example, a few patients could register and get medicine in just 1 min according to our calculation, which was impossible in our hospital

Initially, our analysis recruited 156,635 visits from the information department of the SCMC for the period from August 1, 2019 to January 31, 2020 (Fig. 2, step 1). There were some appointments for which the patients came after a long time following their registration. These visits were excluded from our analysis (Fig. 2, step 2). We also discarded patients who arrived late (Fig. 2, step 3) to prevent such visits from interfering with the results. Because our purpose was to simplify the outpatient process by adjusting the order in which lab tests were performed, patients who did not receive tests were excluded (Fig. 2, step 4). In addition, for the data of some patients, the data on the medicine expenses were missing. This part of data was excluded (Fig. 2, step 5). Similarly, data of patients with illogical discrepancies were also excluded. For example, for a few patients, the data indicated that they registered and received their medicines in just 1 min, which was not feasible (Fig. 2, step 6). We used 1:1 PSM according to the registration time (accurate to minutes). Depending on the results, there was no statistical difference (*p* > 0.05) in the registration time between the two groups after matching.

In another of our studies, to assess the performance of XIAO YI, we invited several doctors to evaluate the recommended tests or examinations by reviewing the chief complaints. These items might be exactly what the patients need, which we define as accuracy. We obtained the data from the hospital information system, and the senior doctors with rich clinical experience judged whether the items ordered by XIAO YI were exact the items the patient needed according to the chief complaint. After preliminary analysis, the accuracy of XIAO YI was 0.92.

### Demographic characteristics of the subjects

Our final dataset comprised 12,342 visits. Among them, 6171 belonged to the conventional group and 6171 belonged to the AI-assisted group. The summary statistics are as follows: for the conventional group: 3298 males, 2873 females, and mean age: 4.57 ± 3.16 years; for the AI-assisted group: 3266 males, 2818 females, and mean age: 3.99 ± 2.87 years. The sex ratio was similar in both groups (*P* > 0.05). Although the difference in children’s ages was significant (*p* < 0.05), we did not consider it as a confounding factor that would affect the results. The majority patients of AI-assisted group (97.68%) and conventional group (89.74%) went to the pediatric internal department for treatment (*p* < 0.05). During that period, few patients visited the gastroenterology (4.12% for controls and 0.16% for cases) or respiratory (6.14% for controls and 0.75% for cases) departments. Because of manual data entry errors, the registration department and birth date of 87 AI-assisted patients had been missing. The detailed information about the patients’ sex, age, and medical department is shown as Table 2.

**Table 2:** Characteristics of the visits

Characteristics	Overall *N* = 12,342	AI-assisted group		Conventional group		χ^2^	*P*
		*N* = 6171	%	*N* = 6171	%		
**Sex**							
Male	6564	3266	52.93	3298	53.44	0.07	**0.80**
Female	5691	2818	45.67	2873	46.56		
Data Missing ^*^	87	87	1.40	0	0		
**Medical department**							
Internal department	11,566	6028	97.68	5538	89.74	506.60	**< 0.01**
Respiratory department	425	46	0.75	379	6.14		
Gastroenterology department	264	10	0.16	254	4.12		
Data Missing ^*^	87	87	1.41	0	0		

^*^ Due to manual data entry errors, this part of data was lost and could not be exported

*AI* Artificial Intelligence

### Comparison between case group and control group

To reiterate, the waiting time was defined as the time from registration to preparation for a laboratory test or examination, and the total cost included the registration fee, test fee, examination fee, and drug fee. As shown in Table 3, for the AI-assisted group, the median waiting time was 0.38 h compared with 1.97 h for the conventional group. The difference was statistically significant (*p* < 0.05). The expenses of the AI-supported group were lower in terms of total cost (*p* < 0.05).

**Table 3:** Efficiency and cost between AI-assisted group vs. Conventional group in pediatric outpatients

	AI-assisted group	Conventional group	Z	*P* ^a^
	Median (P_25_, P_75_)	Median (P_25_, P_75_)		
Waiting time (h) ^b^	0.38 (0.20, 1.33)	1.97 (0.76, 3.48)	−48.40	**< 0.01**
Registration fee (CNY)	25.00 (25.00,25.00)	25.00 (25.00, 40.00)	−22.16	**< 0.01**
Test fee (CNY)	85.00 (65.00,170.00)	85.00 (65.00,190.00)	−9.81	**< 0.01**
Examination fee (CNY)	0.00 (0.00, 0.00)	0.00 (0.00,60.00)	−19.51	**< 0.01**
Drug fee (CNY)	185.43 (106.08, 263.05)	163.40 (78.00, 247.16)	−9.01	**< 0.01**
Total cost (CNY) ^c^	335.97 (244.80, 437.60)	364.58 (249.70, 497.76)	−11.26	**< 0.01**

^a^ P values were calculated by Wilcoxon signed rank tests (for abnormal distribution) or paired Student test (for normal distribution)

^b^ Waiting time defined as the time from registration to preparation for test or examination

^c^ Total cost including the registration fee, test fee, examination fee and drug fee

*AI* Artificial Intelligence, *CN* Chinese Yuan

Because the number of patients in each department was different, we subdivided the patients into 6 subgroups according to the departments. As shown in Table 4, there were more AI-assisted patients in the internal department, while AI-assisted patients in the respiratory and gastroenterology departments were significantly less than those in the conventional group. However, in all departments, we could see that the median waiting times in the AI-assisted group was lower than that in the conventional group (*p* < 0.05).

**Table 4:** Efficiency and total cost of internal department, gastroenterology department, and respiratory department

	Internal Department		Gastroenterology Department		Respiratory Department	
	AI-assisted group	Conventional group	AI-assisted group	Conventional group	AI-assisted group	Conventional group
Visits (N)	6028	5538	46	379	10	254
Waiting time (h)	0.38 (0.20,1.33)	2.16 (0.82,3.58) *	0.54 (0.17,1.44)	1.03 (0.50,1.76) *	0.33 (0.16,2.02)	1.07 (0.58,1.76) *
Total cost (CNY)	334.87 (243.92,434.30)	357.04 (245.16,474.59) *	405.53 (317.49,680.47)	693.38 (327.37,1192.00)	325.80 (283.74,929.87)	438.48 (286.41,696.67) *

After testing, all the data presented non-normal distribution. The median (inter-quartile range) was used to describe the centralized and discrete trend of the data

* *P* < 0.05

In addition, patients were also subdivided into different subgroups according to the tests/examinations they did. Firstly, the most common examinations were abdominal ultrasound and chest X-ray. The number of other examinations was relatively small, so we only compared the patients who received an abdominal ultrasound or chest X-ray. In Table 5, the waiting time of AI-assisted group was significantly lower than that of conventional group (*p* < 0.05). Secondly, because laboratory tests often overlapped, patients who needed a stool routine test, for example, had to take a blood routine test as well. According to statistics, urine routine test, stool routine test, and influenza A and B virus detection were the most common among all the items. Thus, we compared patients who did blood routine test, urine routine test, stool routine test, or influenza A and B virus detection only once. As shown in Table 6, among different test items, the waiting time was still lower in the AI-assisted group than that of conventional group (*p* < 0.05). No other significant associations were found.

**Table 5:** Visits and waiting times in the AI-assisted and conventional groups for abdominal ultrasound and chest radiograph

	Abdomen ultrasound		Chest radiograph	
	AI-assisted group	Conventional group	AI-assisted group	Conventional group
Visits (*N*)	171	701	737	778
Waiting time (h)	0.35 (0.22,1.41)	1.32 (0.56,2.79) *	0.45 (0.20,1.43)	2.00 (0.82,3.21) *

After testing, all the data presented non-normal distribution. The median (inter-quartile range) was used to describe the centralized and discrete trend of the data

* *P* < 0.05

**Table 6:** Visits and waiting times in the AI-assisted and conventional groups for blood routine, urine routine and detection of influenza A and B virus

	Blood routine		Routine urine		Detection of influenza A and B virus	
	AI-assisted group	Conventional group	AI-assisted group	Conventional group	AI-assisted group	Conventional group
Visits (N)	4674	4552	1102	151	195	366
Waiting time (h)	0.38 (0.20,1.33)	2.10 (0.81,3.55) *	0.37 (0.18,1.26)	1.94 (0.91,3.27) *	0.37 (0.22,1.53)	2.37 (0.85,3.78) *

After testing, all the data presented non-normal distribution. The median (inter-quartile range) was used to describe the centralized and discrete trend of the data

* *P* < 0.05

## Discussions

In this study, we verified that with help of XIAO YI, getting a laboratory test or an imaging examination prior to consult a doctor could significantly reduce patients’ waiting time. We also found that accepting the tests or examinations recommended by the AI-assisted system did not result in higher costs; on the contrary, the cost was less than that of ordinary patients. This research suggests a way to improve the outpatient service to a certain extent by reducing the links in the whole process. The number of outpatients in public tertiary general hospitals has increased dramatically. Long waiting time can lead to patients with potentially urgent problems not receiving timely treatment [20]. It may also lead to cancelling or no-show appointments [20, 21]. In other studies, the average waiting time at Chinese general tertiary hospitals was 23 min [2]. The waiting time for outpatient service in pediatric hospitals was considered to be generally longer at 42 min [22]. In our study, as the waiting time was defined as the time from registration to preparation for the examination or test. With AI, the waiting time was reduced to 0.38 h (i.e., < 5 min) from about 2 h before in our hospital.

The waiting time of outpatients has always been a matter of serious concern in China and other developing countries. Substantial research has shown that evaluating and redesigning outpatient systems in the healthcare process would successfully reduce waiting times and improve satisfaction. Studies at tertiary general hospitals in China have reported similar findings. For example, Wang et al. reported that staff carried out a quality circle-themed activity, which reduced the time for patients to see a doctor [23]. Chen et al. suggested that waiting time could be diminished by the introduction of an appointment system and flexible, demand-oriented doctor scheduling according to the number of patients waiting at different times of the workday [24]. However, for pediatric hospitals with a limited number of doctors, it would undoubtedly increase the daily work burden of doctors. In addition, pediatric hospitals and general hospitals are different in many ways. The immune functions of children are still developing, and a variety of diseases caused by climate factors has a significant impact on the number of pediatric visits. Therefore, it is debatable whether the advantages of redesigned outpatient systems are applicable to a large children’s hospital. Based on our results, we believe that an AI-based system would simplify the pediatric outpatient process and decrease the waiting time of patients without increasing (or even reducing) doctors’ workload in a children’s hospital. In the emergency department and the radiology department, there was a precedent for using AI to reduce outpatient time. Curtis [12] investigated the applicability of machine learning models to predict waiting times at a walk-in radiology facility (for radiography) and delay times for scheduled radiology services (CT, MRI, and ultrasound). Accurately predicting waiting times and delays in scheduled appointments might enable staff members to more accurately respond to patient flow. In Lin’s study [25], supervised machine learning models provided an accurate patient wait time prediction and were able to identify the factors with the largest contribution to patient wait times. It is important to emphasize that patient satisfaction increases when patients are told about their expected wait time. Analogous results have been reported in other studies [9, 26–30].

To our knowledge, ours is the first study to use AI for assisting the outpatient process by predicting whether a lab test or an imaging examination is recommended prior to seeing a doctor. The innovation of our study lies in the embedding of the combination of AI-assisted diagnosis and prescription into the outpatient procedure. By extending this system, it is conceivable that the parents of the children could complete a series of steps, such as registration, pre-consultation, and prescription at home or on the way to the hospital with the help of XIAO YI. After registration, patients could immediately undergo the required examination or tests, which considerably increases the efficiency of medical care. Since implementation of the XIAO YI system in 2018, it has assisted more than 270,000 visits, in total, and more than 60,000 children have experienced the new outpatient service. All the datasets we used for training and validation were from patients with real therapeutic experience, and they were more reflective of the real world than recruiting volunteers to participate in the experiment. As in the real world, a patient’s medical process is often subject to change. In addition, a patient’s waiting time is affected by a number of factors, and the most obvious one is the time of registration. Seasons, holidays, and periods of time may affect the flow of patients. Another advantage of this study is that PSM was used to effectively equalize covariates between the nonrandom study groups.

This study contains the following limitations. First of all, the proportion of patients in the AI-assisted group was different in three departments. But in fact, during the study, uniformly trained volunteers and nurses in all three departments were unbiased educating patients about the latest technology and teaching them how to use XIAO YI. Apparently, patients from internal department were more receptive to XIAO YI. The difference might be due to patients’ individual choices. Internal department generally treated patients with common diseases, such as colds, coughs, gastroenteritis, and urinary tract infections. Most of these conditions, which everyone had one or more times in their lives, were not fatal or intractable. So, children’s guardians were more inclined to try XIAO YI when there were too many people in line. But in gastroenterology or respiratory department, things might be complicated, such as unexplained abdominal pain, jaundice, asthma, and tuberculosis. In these cases, the guardians might have insufficient trust in AI technology and prefer to seek help from the real doctors. Nevertheless, it was not a contradiction. According to previous data, there were far more patients from internal department in the same period than in the gastroenterology or respiratory departments. At the same time, most of the internal patients’ conditions were simple, and the diagnoses were also clear. So, the target population of XIAO YI was precisely this kind of patients. Letting them check-up before seeing a doctor not only reduced the waiting time, but also relieved the doctor’s workload. In future, as algorithms maturate and people become more acceptable to AI, XIAO YI will recommend more tests/examinations.

Second, the system was designed for the target patients, that is, patients who needed an imaging examination or a lab test. The patients who did not undergo an examination or test were excluded. Third, the AI system and the hospital information system had to be connected by the unique outpatient number to make the data exchange. If the doctor forgot to enter the patient’s outpatient number during consulting, there would be no way to connect this part of the data. This resulted in missing data and the appearance of illogical values. With debugging and other interventions, this issue can be solved.

Chinese public hospitals, especially the tertiary hospitals, have strong similarities in having an overload of patients and shortage of doctors. Therefore, they all may become applicable scenarios for XIAO YI and benefit from it. As a matter of fact, influenced by the successful experience of the SCMC, other hospitals in Shanghai Pudong New Area have already introduced XIAO YI to ease the work burden of doctors. In the near future, from a polycentric perspective, we will focus on using AI to help patients receive more efficient, accurate, and fair guidance and to reasonably triage patients according to their diseases and the examinations they need.

## Conclusions

In this study, waiting times were significantly reduced in AI-assisted outpatient service process. AI can not only improve medical service but also potentially play a transformative role in the design of processes for enhancing the patient flow.

## Data Availability

The data that support the findings of this study are available from the authors upon reasonable request and with permission of the Shanghai Children’s Medical Center.

## Abbreviations

AI: Artificial intelligence
SCMC: Shanghai Children’s Medical Center
PSM: Propensity score matching
SD: Standard deviation
IQR: Inter-quartile range
